# Comparative perspectives on the Japanese transitional care system

**DOI:** 10.1002/jgf2.732

**Published:** 2024-09-26

**Authors:** Rio Ohashi, Mayuko Sato, Kenzo Takahashi, Hayase Hakariya

**Affiliations:** ^1^ Academic Research Club KADOKAWA DWANGO Educational Institute Okinawa Japan; ^2^ Institute for Pharmaceutical and Social Health Sciences Ise Japan; ^3^ Teikyo University Graduate School of Public Health Tokyo Japan; ^4^ Tetsuikai Research Institute Tokyo Japan; ^5^ Interfaculty Institute of Biochemistry, University of Tuebingen Tuebingen Germany

To the Editor,

The Japan Pediatric Society (JPS) recommended transitional care for childhood‐onset chronic diseases in 2022.^1^ Advances in pediatric medicine have enabled the effective treatment and control of previously incurable diseases. However, this progress led to pediatricians continuing to treat adult patients for prolonged treatments and follow‐ups. This situation extends the workload for pediatricians and involves managing adult diseases beyond their specialty. Therefore, a comprehensive support system is essential to ensure the smooth transition of patients to appropriate adult medical providers as they age. Herein, we compare the approaches to transitional care in Japan and the United Kingdom (UK), both committed to transitional care support. We selected the UK for comparison due to its pioneering role in transitional care support. The UK provides comprehensive support in each local health authority under the lead of the National Health Service (NHS). Given that Japan has recently begun implementing support on a prefectural basis, there might be potential to adapt and apply strategies from the UK system to the Japanese context.

In the UK, the NHS emphasizes support throughout the transitional care process, from initial planning to transfer and subsequent support in adult care.^2^ Pediatric consultants assist patients with neonatal diseases, while nurses in the transition team coordinate sharing medical records with general practitioners. This structured approach in the UK differs from that in Japan. The JPS defines transitional care as a “transition from pediatric care to adult care appropriate for individual patients.”^1^ Thus, the JPS advocates for supporting medical systems rather than overseeing the entire transition process.

One background for the JPS's focus presumably stems from the delayed establishment of transitional care support centers in Japan with function of bridging pediatric care specialists and adult care facilities supporting patients in transition. Despite the enactment of the Basic Law for Child and Maternal Health and Child Development in 2018, Japan's Ministry of Health, Labour and Welfare requested prefectures to establish these centers, as of January 2024,^3^ only 9 out of 47 prefectures had done so, primarily in urban areas such as Tokyo and Osaka. Given that 120,000 individuals receive annual subsidies for medical expenses related to chronic childhood‐onset diseases,^4^ additional efforts are needed to accelerate the establishment of centers and address regional disparities.

Moreover, the nine Japanese transitional care support centers exhibit functional and administrative disparities. Different prefectures assign various departments to oversee transitional care support, leading to inconsistencies due to the lack of standard requirements. For example, some prefectures assign child support departments as in Kanagawa and Tokyo, while others (*n* = 7) assign healthcare and medical services. This lack of uniformity may confuse patients seeking transitional healthcare support across prefectural borders.

Conversely, the NHS in the UK directs local health authorities to provide consistent support systems. They assess individual care needs based on patient‐prepared statements^5^ and may offer mental health support through the child and adolescent mental health service. This approach ensures a smoother transition through the formation of personalized and specialized teams.^5^ However, a shortage of human resources is a fundamental issue in maintaining uniform and high‐quality care, leading to burnout among medical professionals and frequent strikes.

In conclusion, Japan should establish transitional care support centers across all prefectures to ensure equal transitional care support. Although medical systems and resources vary among regions, the Japanese government can take a flagship for standardization of requirements for establishing centers. Additionally, disseminating this issue more broadly among patients and adult clinics in Japan is crucial. Indeed, the recognition of transitional care support centers is low, at 11.2% among parents of pediatric patients, according to the survey conducted by the center in Saitama Prefecture.^6^ Whereas, no studies have assessed pediatricians' perception of these centers to the best of our knowledge, which may imply the need for continuous promotion and regular evaluation of their recognition. Adult clinics should be prepared to receive patients who have relied solely on pediatricians. To achieve this, we propose equipping the transitional care support centers with educational roles for adult clinics. Sharing patient information between pediatricians and adult clinics could lead to more effective transitional care support in Japan. In this regard, expanding financial incentives to enhance the workforce, such as involving more medical social workers, who are responsible for individual patients, could be beneficial.

## AUTHOR CONTRIBUTIONS

R.O. and H.H. conceptualized the work, collected the information required for the project, and wrote the original manuscript. M.S. and K.T. collected and provided further fruitful information, reviewed the original draft critically, and discussed and interpreted the contents. HH supervised the project. All authors read through the submitted manuscript and approved to be published.

## CONFLICT OF INTEREST STATEMENT

H.H. receives personal fees from Kadokawa Dwango Educational Institute. M.S. is employed by Ono Pharmaceutical Company Ltd., outside the submitted work. R.O. and K.T. have no financial interests to disclose.
